# Antiviral Metal Complexes

**DOI:** 10.1155/MBD.1997.173

**Published:** 1997

**Authors:** Erik De Clercq

**Affiliations:** Rega Institute for Medical Research Katholieke Universiteit Leuven Leuven B-3000 Belgium

## Abstract

The initial events (virus adsorption and fusion with the cells) in the replicative cycle of human immunodeficiency virus (HIV) can serve as targets for the antiviral action of metal-binding compounds such as polyanionic compounds (polysulfates, polysulfonates, polycarboxylates, polyoxometalates, and sulfonated or carboxylated metalloporphyrins), bicyclams and G-octet-forming oligonucleotides. The adsorption and fusion of HIV with its target cells depends on the interaction of the viral envelope glycoproteins (gp 120) with the receptors (CD4, CXCR4) at the outer cell membrane. We are currently investigating how the aforementioned compounds interfere with these viral glycoproteins and/or cell receptor.


### ANTIVIRAL METAL COMPLEXES

Erik De Clercq

*Rega Institute for Medical Research, Katholieke Universiteit Leuven, B-3000 Leuven, Belgium*

### Abstract

The initial events (virus adsorption and fusion with the cells) in the replicative cycle of human immunodeficiency virus (HIV) can serve as targets for the antiviral action of metal-binding compounds such as polyanionic compounds (polysulfates, polysulfonates, polycarboxylates, polyoxometalates, and sulfonated or carboxylated metalloporphyrins), bicyclams and G-octet-forming oligonucleotides. The adsorption and fusion of HIV with its target cells depends on the interaction of the viral envelope glycoproteins (gp120) with the receptors (CD4, CXCR4) at the outer cell membrane. We are currently investigating how the aforementioned compounds interfere with these viral glycoproteins and/or cell receptor.

### Introduction

There are ten steps in the replicative cycle of human immunodeficiency virus (HIV) that could be considered as targets for chemotherapeutic interventions (Table 1) (1). The early events in HIV infection, i.e. virus adsorption to the cells and virus cell fusion, have been shown to be the points of attack for some metal complexes or organic compounds containing metals. Here I will discuss those compounds among the metal complexes that interfere with the virus adsorption and/or virus cell fusion (i.e. polyanionic substances, bicyclam derivatives and G-octet-forming oligonucleotides).

Table 1. HIV replicative cycle

| | |
|---|---|
| 1. | Virus adsorption to the cells |
| 2. | Virus-cell fusion |
| 3. | Virus uncoating |
| 4. | Reverse transcription |
| 5. | Proviral DNA integration |
| 6. | Proviral DNA replication |
| 7. | Proviral DNA transcription to viral mRNA |
| 8. | Viral mRNA translation to viral precursor proteins |
| 9. | Maturation (proteolysis/myristoylation/glycosylation) |
| 10. | Budding (assembly/release) |

The key molecule in the viral adsorption/fusion process is the viral envelope glycoprotein gp120 (Fig. 1), which has a highly convoluted structure containing several regions referred to as variable regions such as V3 and V4, which are assumed to interact with the corresponding receptors at the host cell surface.

**Polyanionic substances**

Foremost among the polyanionic substances that have been shown to interact with the binding of HIV to the cells are the polysulfates such as dextran sulfate, dextrin sulfate, curdlan sulfate, pentosan polysulfate, mannan sulfate, sulfoevernan, fucoidan, polyacetylal polysulfate (PAPS) and polyvinylalcohol sulfate (PVAS) (Fig. 2) (2). These compounds inhibit HIV-induced cytopathicity in cell cultures at an 50% effective concentration ($EC_{50}$) of about 0.1 to 1 µg/ml, that is at a concentration which is 1,000- to more than 10,000-fold lower than the concentration exhibiting cytotoxicity [50% cytotoxic concentration ($CC_{50}$)] (Table 2).

In addition to the polysulfates, polysulfonates such as suramin, Evans blue, bis(naphalenedisulfonate), polystyrene sulfonate (PSS), and polyvinyl sulfonate (PVS) (Fig. 3) and polycarboxylates [in particular polymerized aurintricarboxylic acid (ATA)] (Fig. 4) have also been shown to inhibit HIV replication with $EC_{50}$ values similar to those of the polysulfates (1).

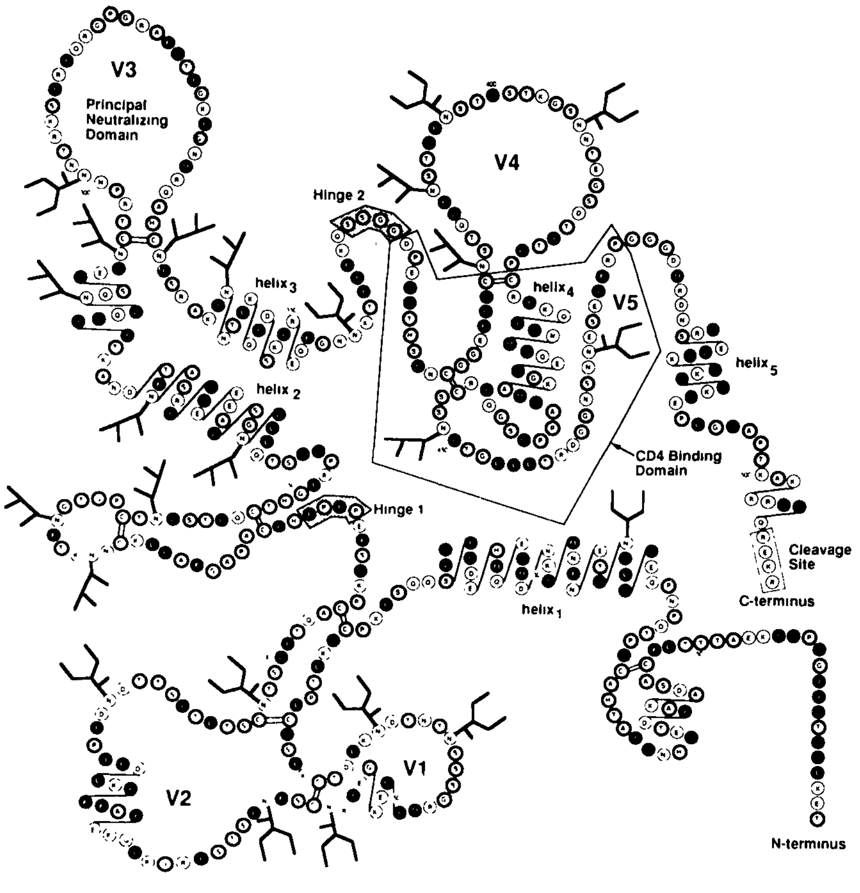

Fig. 1. The HIV-1 envelope (surface) glycoprotein gp120

A large series of polyoxometalates (Fig. 5), i.e. JM1590, which corresponds to $K_{13}[Ce(SiW_{11}O_{39})_2].26H_2O$, are known to inhibit HIV replication at an $EC_{50}$ of 0.3 to 3 µg/ml with $CC_{50}$ values higher than 100 µg/ml (Table 3). Akin to the polysulfates and polysulfonates, the polyoxometalates inhibit the HIV replicative cycle at the level of virus adsorption to the cells (3).

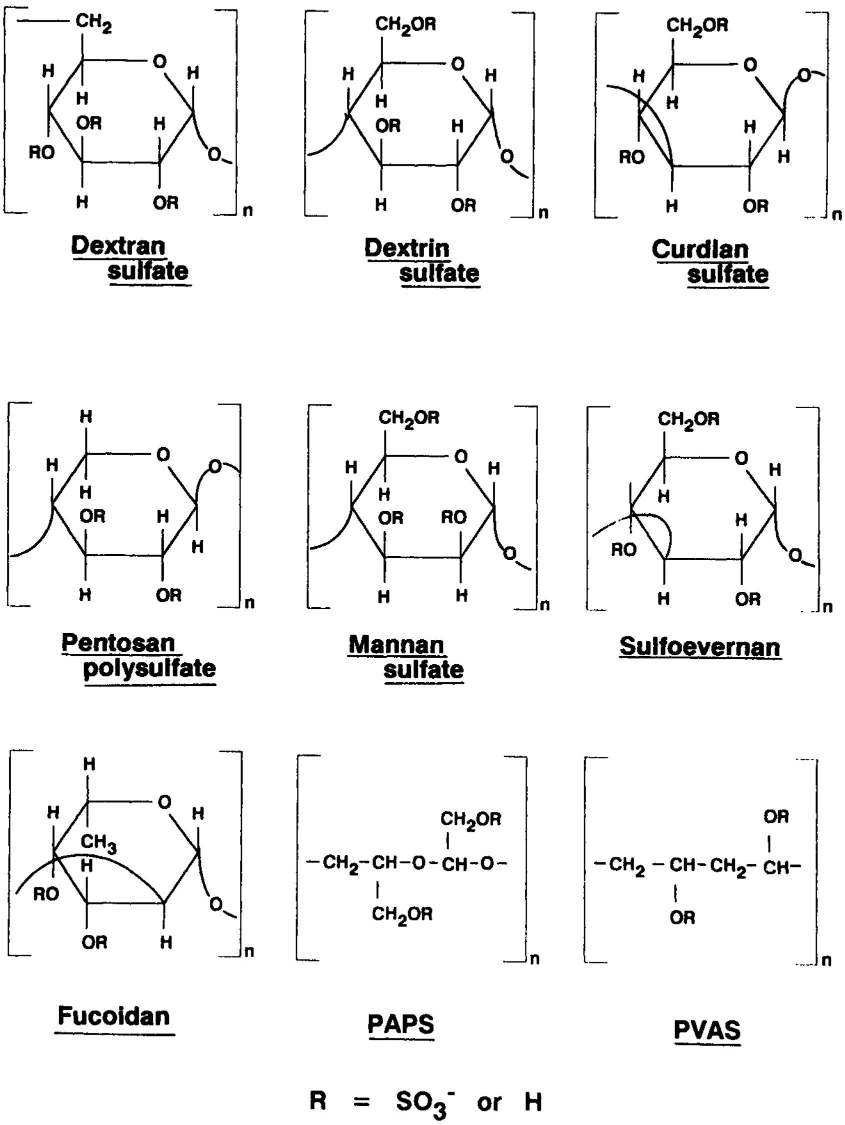

Fig. 2. Sulfated polysaccharides and polymers

A new class, that of the anionic (sulfonated or carboxylated) metalloporphyrins, have been recently shown to inhibit HIV replication (Table 4) (4). Metals (such as Fe or Ni) play an important role in the selectivity of the compounds, for their presence significantly reduces cytotoxicity while maintaining antiviral activity (Table 4).

An interesting feature of the polyanionic substances is that their antiviral activity is not limited to HIV but also extends to various other enveloped viruses such as herpesviruses [herpes simplex virus (HSV), cytomegalovirus (CMV)], influenza A virus, respiratory syncytial virus (RSV), arenaviruses (Junin virus, Tacaribe virus) and rhabdoviruses [such as vesicular stomatitis virus (VSV)] (Fig. 6). This broad spectrum antiviral action considerably enhances the therapeutic potential of these compounds for the treatment of viral diseases.

Table 2. Inhibitory effect of sulfated polysaccharides on the cytopathicity of HIV-1($III_B$) in MT-4 cells

| Compound | $EC_{50}$ (μg/ml) | $CC_{50}$ (μg/ml) | Selectivity index ($CC_{50}/EC_{50}$) |
|---|---|---|---|
| Dextan sulfate (MW 5000) | 0.5 | > 2500 | > 5000 |
| Dextrin sulfate (MW 3000) | 2.1 | > 500 | > 238 |
| α-Cyclodextrin dodecasulfate | 6.5 | > 2500 | > 385 |
| β-Cyclodextrin tetradecasulfate | 0.8 | > 2500 | > 3125 |
| γ-Cyclodextrin hexadecasulfate | 0.2 | > 2500 | > 12500 |
| Pentosan polysulfate (MW 3100) | 0.19 | > 2500 | > 13150 |
| Fucoidan | 1.4 | 1060 | 757 |
| Heparin (MW 11000) | 0.58 | > 2500 | > 4310 |
| λ-Carrageenan | 0.54 | > 625 | > 1157 |
| Mannan sulfate (MW 30000) | 1.2 | > 2500 | > 2083 |
| Sulfated E.coli K5 glycan | 0.67 | 260 | 388 |
| Periodate-treated heparin | 0.52 | > 2500 | > 4807 |
| Polyacetal polysulfate (MW 30000) | 0.4 | > 2500 | > 6250 |
| Polyvinylalcohol sulfate (MW 20000) | 0.18 | > 2500 | > 13800 |

We have recently succeeded in obtaining mutants resistant to polyanionic substances after passaging HIV in the presence of dextran sulfate (5). It thus appears possible for the virus to develop resistance to these polyanionic substances (Fig. 7). The resistance mutations appear to be located predominantly in the V3 loop of the gp120 glycoprotein (Fig. 8) and render the overall charge of the V3 loop less positive, thus resulting in a diminished electrostatic interaction with the polyanionic compounds.

Polyanionic substances such as polyoxometalates (i.e. polyoxosilicotungstates) inhibit the replication of HIV, HSV and CMV at the virus adsorption step. They inhibit influenza A virus and RSV at the virus-cell fusion step (6).

Little is known about the therapeutic/prophylactic potential of the polyanionic substances in the clinic. Protective effects with these compounds against HSV and influenza A virus infections *in vivo* (mice) have been described with polyoxotungstates and polysulfonates when administered systemically (intraperitoneally) or topically (intranasally), respectively (7,8). In particular, topical administration of the polyanionic compounds, for instance as a vaginal formulation, seems to be an attractive modality for the prevention of sexually transmitted HIV and HSV infections (9).

**Bicyclams**

The bicyclams (10) represent a new class of highly potent and selective HIV inhibitors. They originated from the serendipitous discovery of anti-HIV activity in a monocyclam preparation that contained bicyclam as contaminant. Starting from this lead compound, several bicyclam derivatives were prepared that showed increased anti-HIV activity (Fig. 9) (11). The most active of this series is the bicyclam JM3100 (Fig. 10) which has been found to inhibit HIV-induced cytopathicity at a concentration of a few nanograms per ml, while not being toxic to the host cells at concentrations up to 500 μg/ml, thus achieving a selectivity index of 100,000 or higher (Table 5) (12).

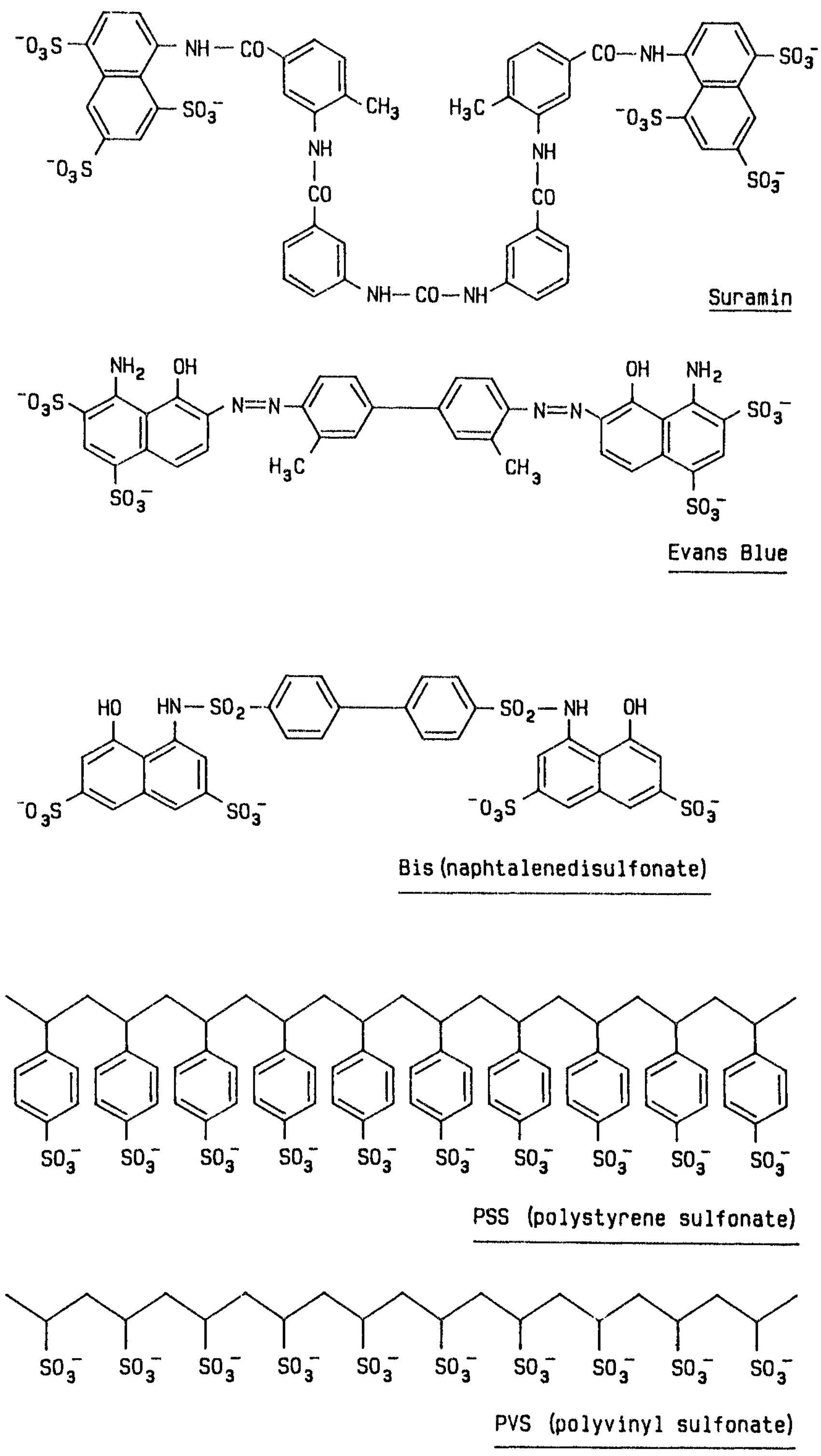

Fig. 3. Sulfonated polymers

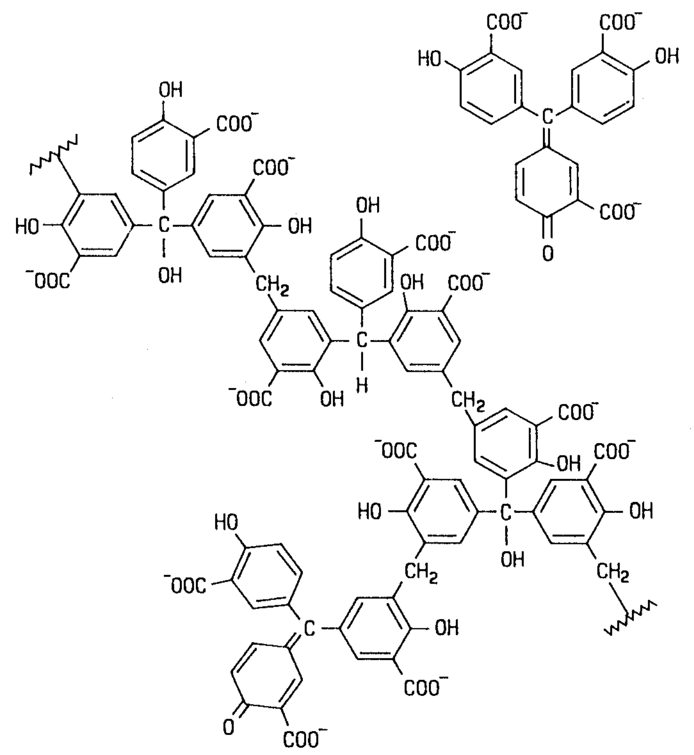

Fig. 4. Polycarboxylates: ATA (aurintricarboxylic acid)

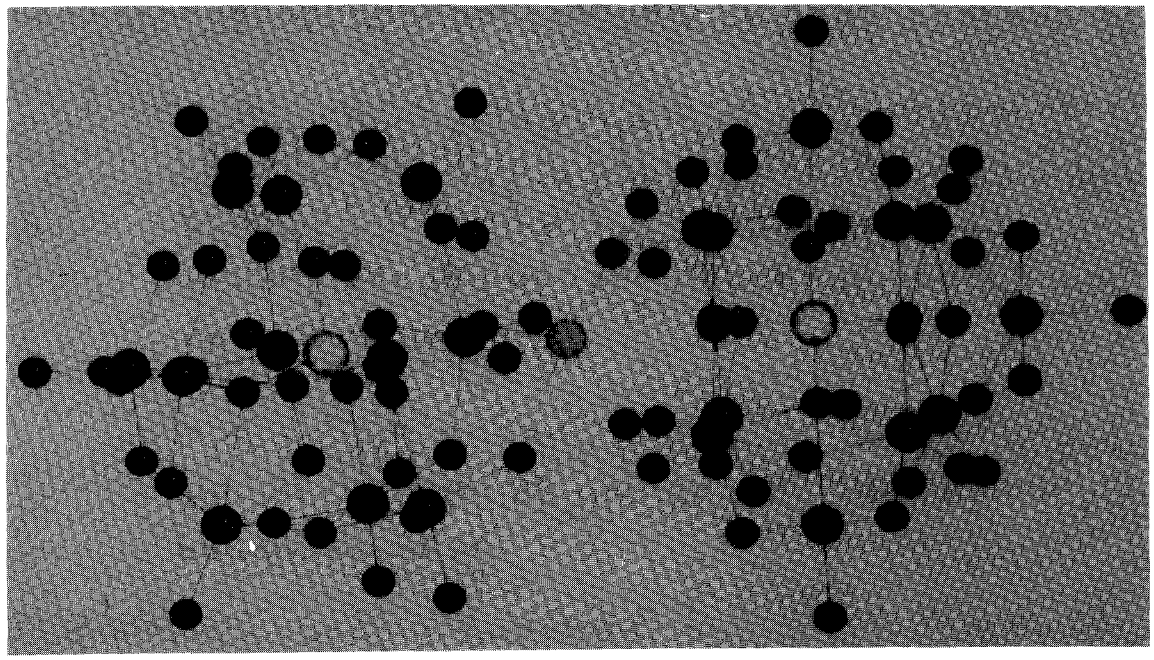

Fig. 5. JM1590: $K_{13}[Ce(SiW_{11}O_{39})_2].26\ H_2O$

Table 3. Anti-HIV activity of polyoxometalates

| Compound | $EC_{50}$ (μg/ml) | $CC_{50}$ (μg/ml) | Selectivity index ($CC_{50}/EC_{50}$) |
|---|---|---|---|
| JM1583 : $K_5[BW_{12}O_{40}]$ | 1.4 | 654 | 467 |
| JM1590: $K_{13}[Ce(SiW_{11}O_{39})_2].26H_2O$ | 0.7 | 230 | 328 |
| JM1591: $K_{12}H_2P_2W_{12}O_{48}.24H_2O$ | 0.3 | 339 | 1130 |
| JM1596: $K_{10}[P_2W_{18}Zn_4(H_2O)_2O_{68}].20H_2O$ | 0.7 | 466 | 666 |
| JM1809: $K_8HP_2W_{15}V_3O_{62}.34H_2O$ | 1.1 | 293 | 266 |
| JM2766: $K_6[BW_{11}Ga(H_2O)O_{39}]$ | 2.8 | > 500 | > 178 |
| JM2815: $K_5[SiW_{11}(C_5H_5)TiO_{39}]$ | 1.9 | > 500 | > 263 |
| JM2820: $[Me_3NH]_8[Si_2W_{18}Nb_6O_{77}]$ | 3.2 | > 500 | > 156 |

Table 4. Anti-HIV activity of metalloporphyrins

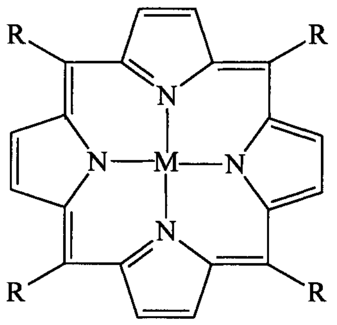

| R | M | $EC_{50}$[a] (μg/ml) | $CC_{50}$[b] (μg/ml) | SI[c] |
|---|---|---|---|---|
|  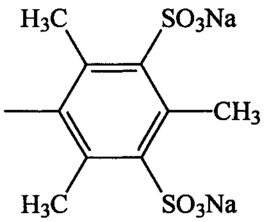 | $H_2$<br>Mn<br>Fe | 1<br>36<br>4 | 4<br>> 100<br>> 100 | 4<br>> 2.8<br>> 25 |
|  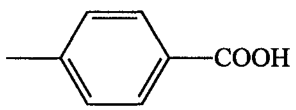 | $H_2$<br>Fe<br>Ni | 0.9<br>0.5<br>1 | 23<br>> 90<br>> 90 | 25<br>> 180<br>> 90 |

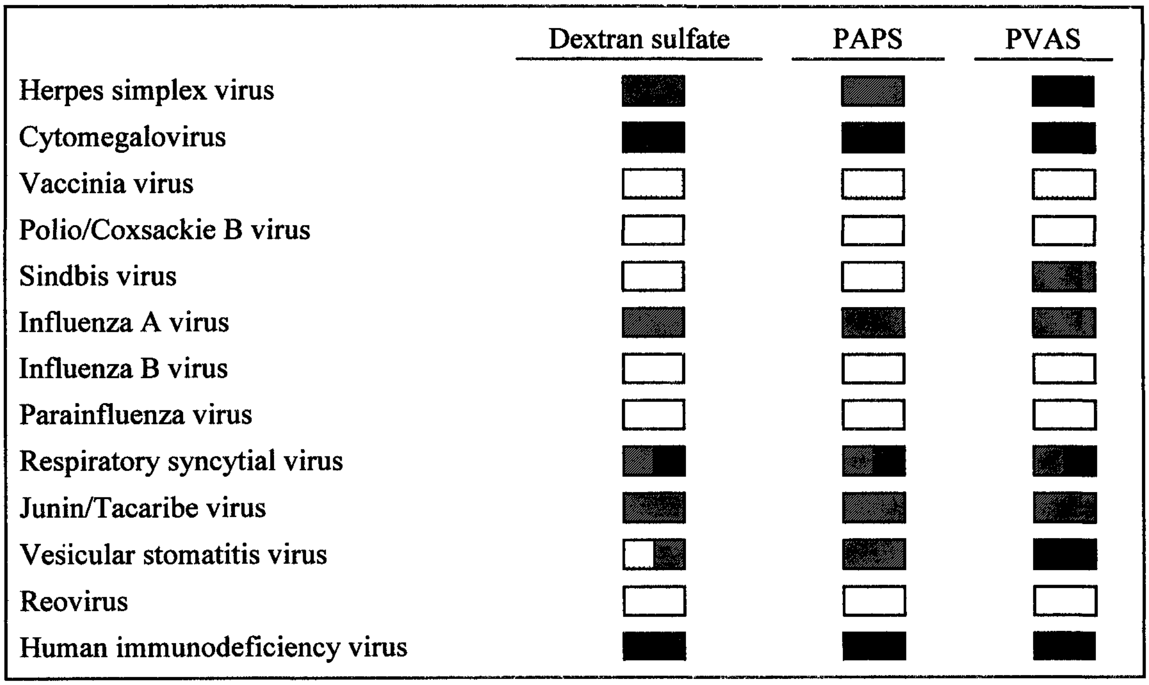

Fig. 6. Antiviral activity spectrum of polysulfates

Minimal effective concentration in the range of 0.1-1 µg/ml (■), 1-10 (µg/ml (▩), 10-100 (µg/ml) (□), or > 100 µg/ml (□)

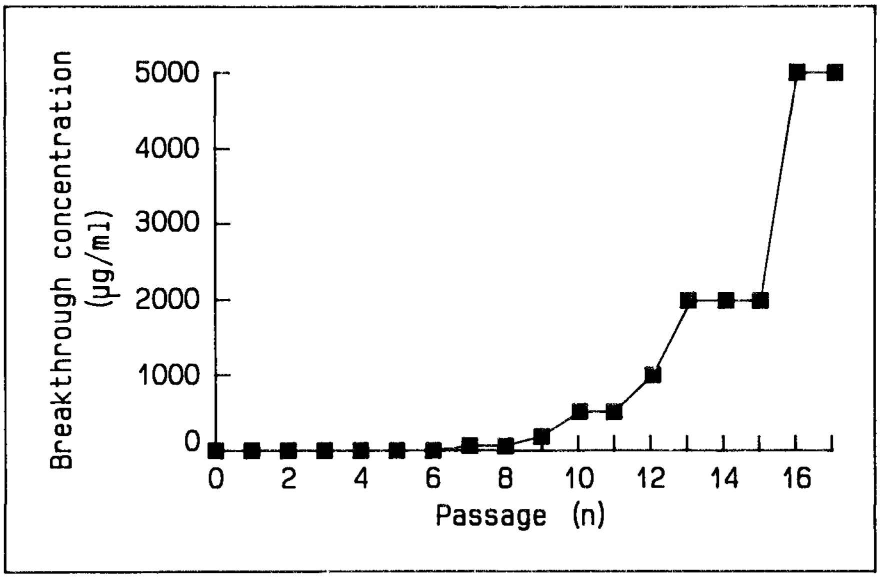

Fig. 7. Rate of resistance development of HIV-1 NL4-3 to dextran sulfate (MW 5000)

MT-4 cells were infected with virus in the presence of 5 x the $EC_{50}$ (passage 0). Every 5 to 6 days supernatant of the cell culture was used to re-infect fresh MT-4 cells in the presence of the same or a 2- to 5-fold higher compound concentration, depending on the cytopathicity observed.

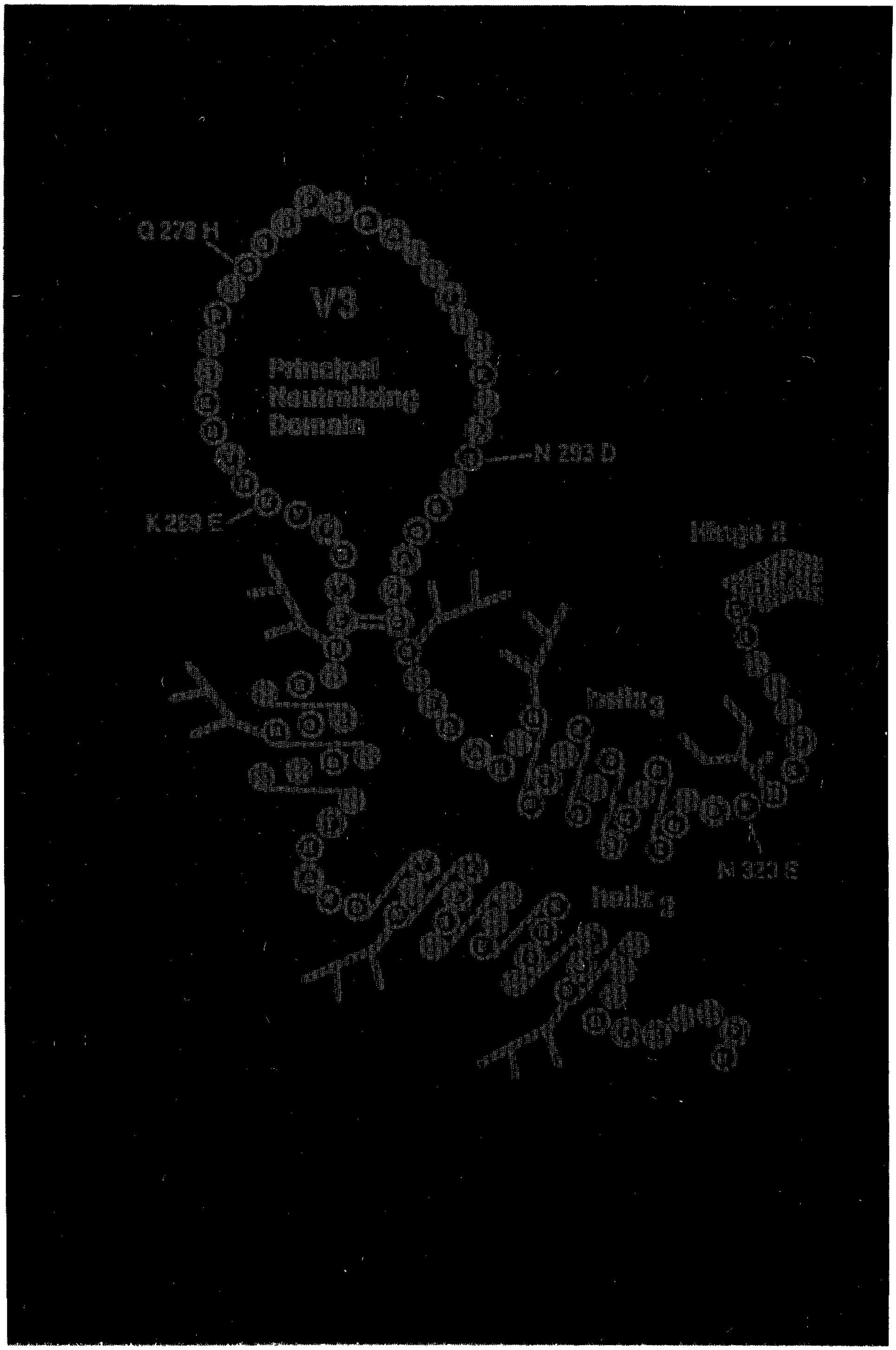

Fig. 8 Portion of the HIV-1 envelope glycoprotein gp120 with the mutations conferring resistance to dextran sulfate

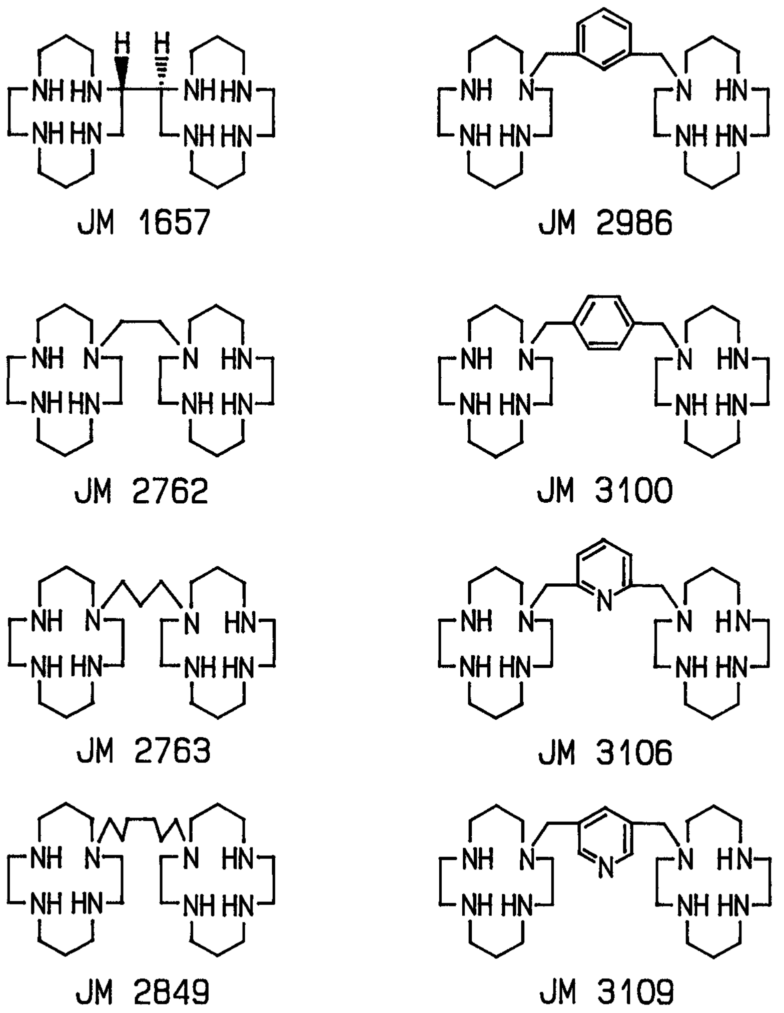

Fig. 9. Bicyclams: bis(1,4,8,11-tetraazacyclotetradecane) derivatives

Time-of-addition experiments indicated that the bicyclams (i.e. JM2763) inhibit the HIV replicative cycle at a time point that is situated between virus adsorption and reverse transcription (Fig. 11). From these time-of-addition experiments we must conclude that the bicyclams interact with the fusion/uncoating process (13). This process involves the removal of the envelope as well as capsid proteins from the viral RNA genome so that the latter can be transcribed by the reverse transcriptase. Theoretically, any of the viral envelope glycoproteins (gp120, gp41) or capsid proteins (p17, p24, p9, p7) could be considered as possible targets for the interaction with the bicyclams (Fig. 12). Originally (13,14), we envisaged the capsid protein p7 (Fig. 13) as a possible target for the bicyclams, as this protein contains two zinc fingers that could possibly make zinc-coordination complexes with the bicyclams.

After painstaking efforts, we succeeded in obtaining mutants that were resistant to the bicyclams JM2763 and JM3100 (Fig. 14) (15,16). Sequence analysis of these mutants revealed the presence of several mutations within the gp120 glycoprotein located in the V3-V4 region (Fig. 15) (17). Although it is not clear yet which and how many of these mutations are required for engendering resistance, it is obvious that the primary site of interaction for the bicyclams is the gp120 rather than any of the other viral glycoproteins or capsid proteins.

The role of several metals in the interaction of the bicyclam JM3100 with HIV has been assessed. From Fig. 16, it is evident that Zn facilitates the binding of the bicyclams to the virus. Equilibrium analysis studies revealed that the optimal binding of JM3100 with the virus is achieved at Zn concentrations of 0.2 to 0.6 mM (Fig. 17). That Zn may play a key role in the anti-HIV activity of the bicyclams is also evident from Table 6. Only the Zn complex with the bicyclam JM3100 (i.e. JM3479) was equipotent to JM3100. The other metal bicyclam complexes (JM3462, JM3469, JM3461 and JM3158) containing Ni, Cu, Co, Pd, respectively, showed gradual loss in activity, the Pd complex being inactive as an anti-HIV compound.

JM3100 has been found efficacious *in vivo*, in decreasing the virus load in the SCID-hu Thy/Liv mice (that is, severe combined immune deficient mice reconstituted with human fetal thymus and liver) infected with HIV (18), and the antiviral efficacy of JM3100 was enhanced when combined with other anti-HIV drugs such as zidovudine (AZT) and didanosine (ddI). This opens interesting perspectives for the clinical use of JM3100 in HIV-infected individuals. Clinical trials with JM3100 (now referred to as AMD 3100) have been planned.

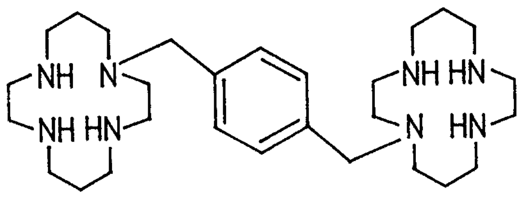

**8 HCl $2H_2O$**

Fig. 10. 1,1'-[1,4-phenylenebis-(methylene)]-bis-1,4,8,11-tetraazacyclotetradecane octahydrochloride dihydrate (JM3100)

Table 5. Anti-HIV activity of bicyclam JM3100

| Virus | Strain | Cell line | $EC_{50}$ (μg/ml) | $CC_{50}$ (μg/ml) | Selectivity index $CC_{50}/EC_{50}$ |
|---|---|---|---|---|---|
| HIV-1 | $III_B$ | MT-4 | 0.005 | > 500 | > 100000 |
| HIV-1 | RF | MT-4 | 0.001 | > 500 | > 500000 |
| HIV-1 | HE | MT-4 | 0.003 | > 500 | > 167000 |
| HIV-2 | ROD | MT-4 | 0.007 | > 500 | > 71400 |
| HIV-2 | EHO | MT-4 | 0.004 | > 500 | > 125000 |
| SIV | MAC251 | MT-4 | > 100 | > 100 | ... |
| SIV | AGM3 | MOLT-4 | > 100 | > 100 | ... |
| SIV | MNDGB1 | MOLT-4 | > 100 | > 100 | ... |

Table 6. Inhibitory effects of different bicyclam-metal complexes on HIV-induced cytopathicity and syncytium formation

| Compound | Complexing metal | $EC_{50}$ (μg/ml) | | | |
|---|---|---|---|---|---|
| | | Viral cytopathicity | | Syncytium formation | |
| | | HIV-1($III_B$) | HIV-2(ROD) | HIV-1($III_B$) | HIV-2(ROD) |
| JM3100 | | 0.009 | 0.021 | 0.1 | 1.8 |
| JM3479 | Zn | 0.008 | 0.025 | 0.1 | 0.2 |
| JM3462 | Ni | 0.017 | 0.028 | 0.3 | 0.3 |
| JM3469 | Cu | 0.048 | 0.21 | 1.5 | 2.4 |
| JM3461 | Co | 9.74 | 18.21 | 62.5 | 125 |
| JM3158 | Pd | 68.62 | > 250 | > 250 | > 250 |

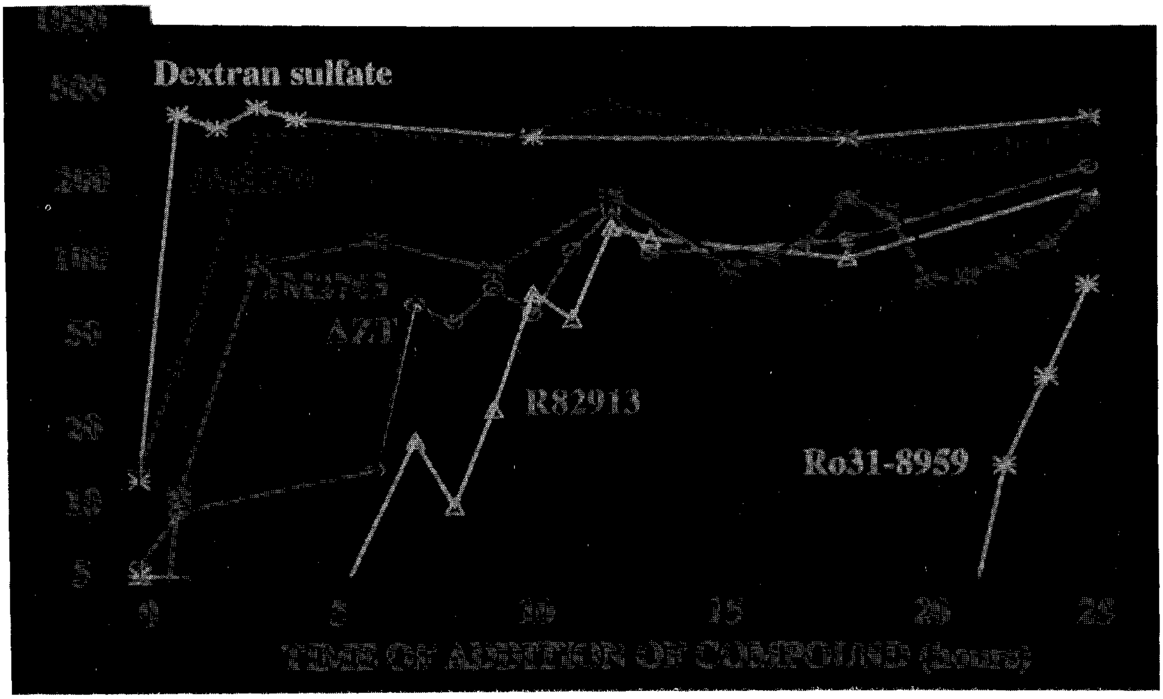

Fig. 11. Time of addition experiment

Compounds dextran sulfate, polyoxometalates JM1590 and JM1657, bicyclam JM2763, AZT, DDI, TIBO R82913 and protease inhibitor Ro31-8959 were added at different times [0, 1, 2, 3, ... hours after infection of MT-4 cells with HIV-1($III_B$)], and viral capsid p24 antigen was measured 29 hours post infection.

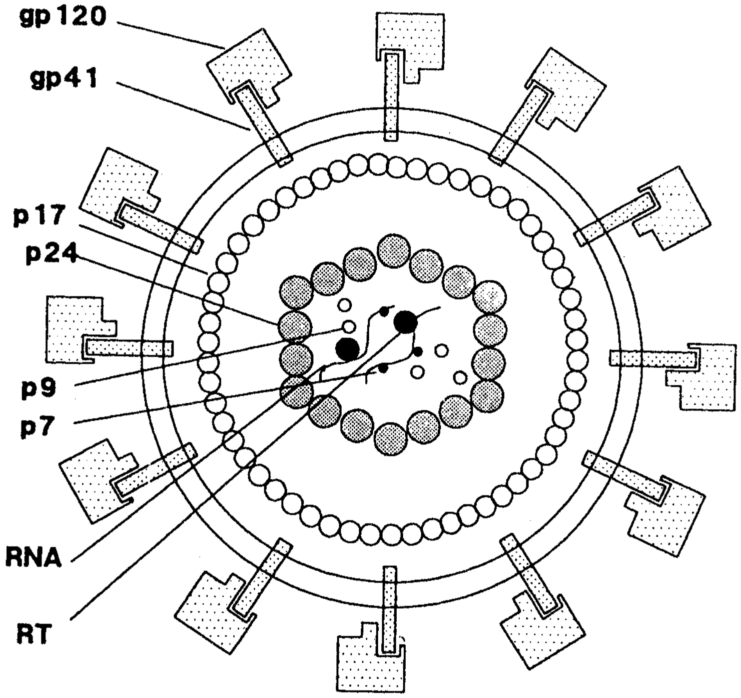

Fig. 12. Schematic cross-section of HIV-1 particle with glycoproteins (gp120, gp41), capsid proteins (p17, p24, p9, p7), RNA genome (2 segments) and reverse transcriptase

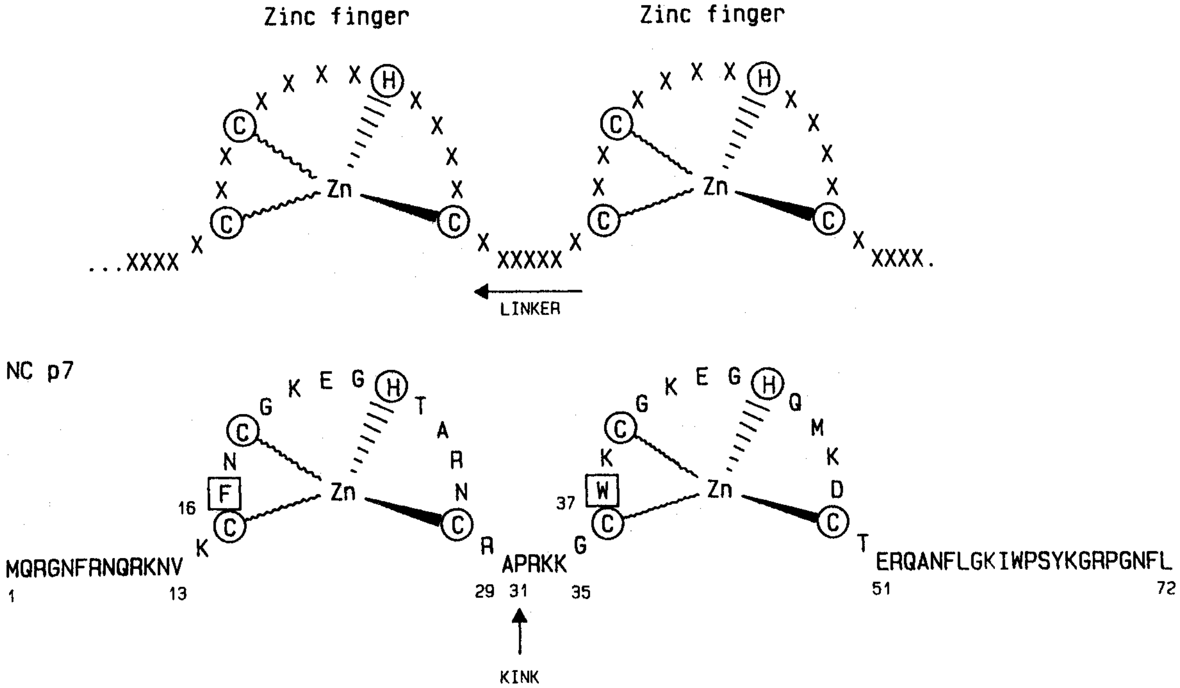

Fig. 13. Nucleocapid protein p7 contains 2 Zn-finger domains

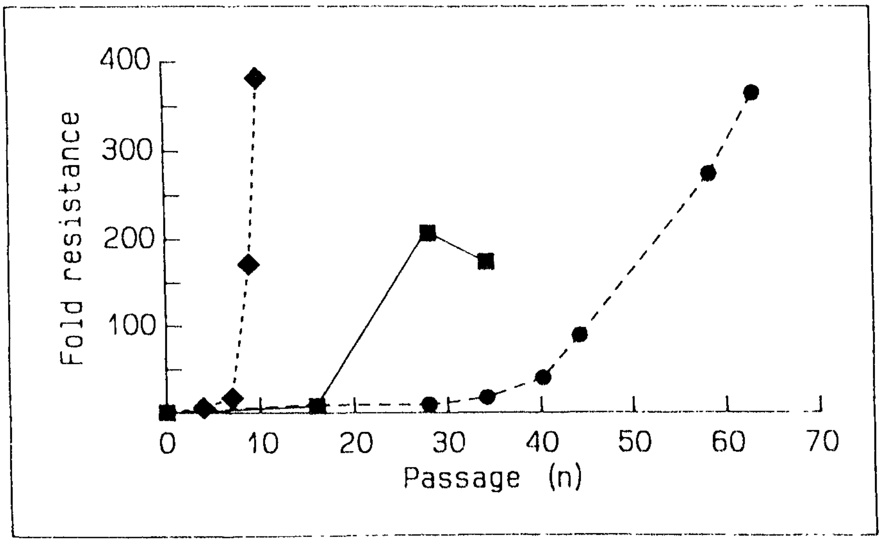

Fig. 14. Rate of resistance development of HIV-1 NL4-3 to the bicyclams JM2763 and JM3100, and to TIBO R86183

At different passages of the virus in MT-4 cells, $EC_{50}$ values were determined and compared with wild-type $EC_{50}$. The ratio of $EC_{50}$ (passage n) to $EC_{50}$ (passage 0) is displayed in function of passage n for JM2763 (■), JM3100 (●) and R86183 (◆).

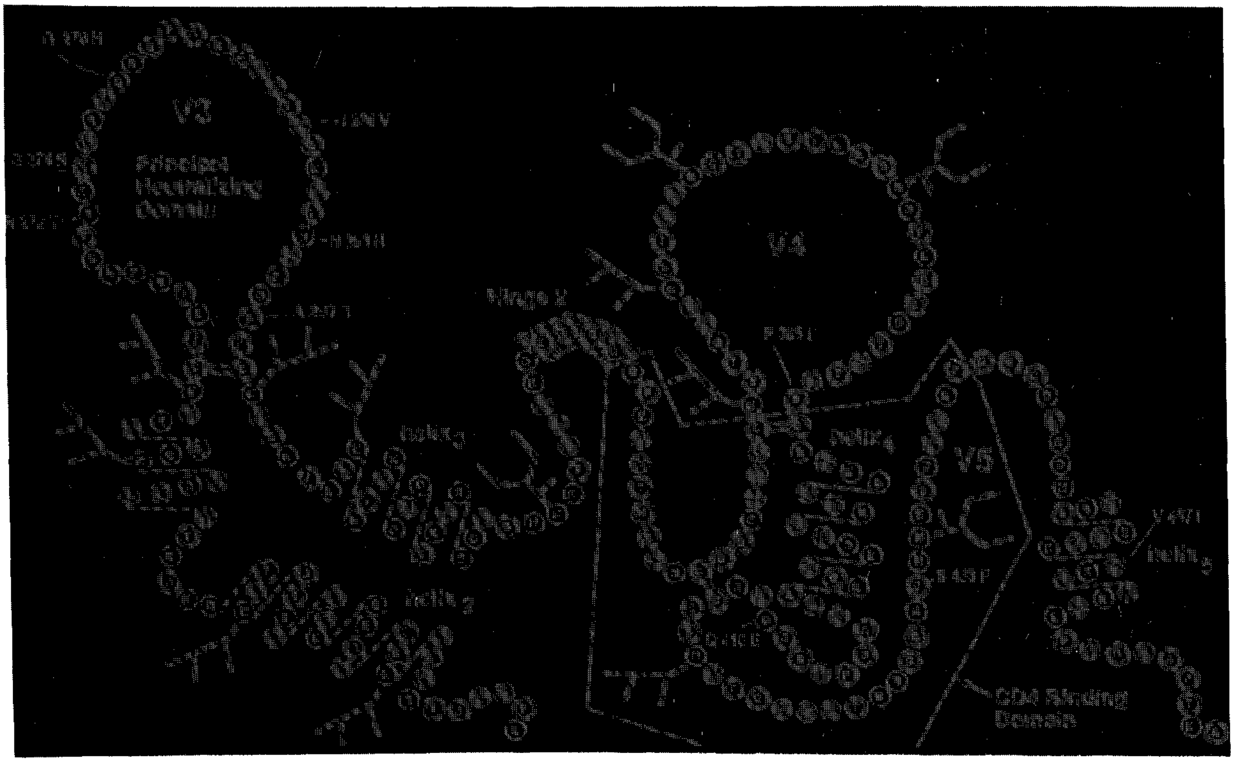

Fig. 15. Portion of the HIV-1 envelope glycoprotein gp120 with the mutations conferring resistance to the bicyclam JM3100

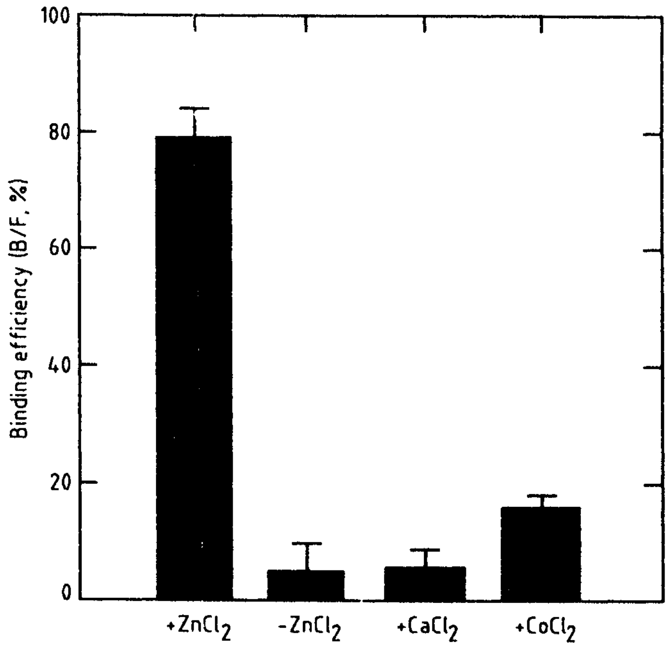

Fig. 16. Equilibrium dialysis binding study (part I)

Binding efficiency of metal-bound (B) versus free (F) bicyclam [$^{14}$C]JM3100 to HIV-1 lysate. Final concentrations: 10 μg/ml (12 μM) for bicyclam; 0.6 mM for $ZnCl_2$, $CaCl_2$ and $CoCl_2$ and 0.4 mg/ml for viral protein. Incubation for 4 hr at 37°C.

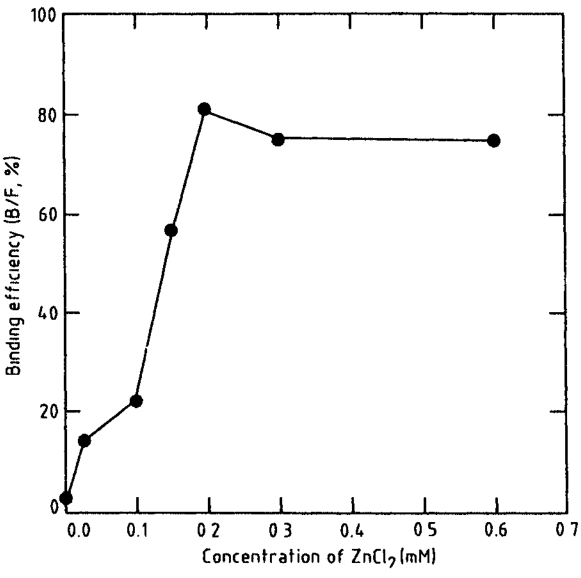

Fig. 17. Equilibrium dialysis binding study (part II)

Binding effficiency of metal-bound (B) versus free (F) bicyclam [$^{14}$C]JM3100 to HIV-1 lysate. Final concentrations: 10 μg/ml (12 μM) for bicyclam; varying concentrations for $ZnCl_2$ and 0.4 mg/ml for viral protein. Incubation for 4 hr at 37°C.

**G-octet-forming oligonucleotides**

The oligonucleotide 5'GTGGTGGGTGGGTGGGT3' which forms a G-octet with potassium in the middle (Fig. 18) (19) has been shown to exhibit activity against different HIV strains in cell culture. As shown in Table 7, the G-octet forming oligonucleotide T30177 (also referred to as AR177 or Zintevir) shows activity against the HIV-1($III_B$) and HIV-1(RF) strain at an $EC_{50}$ of 0.15-2.8 and 0.03-0.3 μM, respectively (20). The anti-HIV activity of T30177 in cell culture persists for several weeks after an initial 4-day exposure of the cells to the compound and subsequent removal of the drug. This contrasts with the behavior of other anti-HIV compounds which rapidly loose their activity when removed after the original 4-day exposure (Fig. 19).

It has been shown that the T30177 inhibits the HIV-1 integrase (Fig. 20). The HIV DNA integration is an highly complicated process involving at least 3 steps (endonuclease, strand-transfer and DNA ligation) (Fig. 21) and the T30177 would interfere with the first step (endonuclease) of the integration process (21,22).

However, it is doubtful that the inhibitory effect of T30177 on the HIV integrase would account for the anti-HIV activity observed with the compound in cell culture experiments, as time-of-addition experiments with T30177 have indicated that the compound inhibits HIV replication at a step which coincides with virus adsorption and/or fusion (Fig. 22). Also, resistant HIV strains selected under continuous pressure of T30177 revealed the presence of mutations in the gp120 molecule, but not in the integrase gene (23), again pointing to the viral adsorption/fusion process as the primary target for the anti-HIV action of T30177.

Clinical studies with zintevir (T30177) have been initiated. When administered intravenously as single- or repeat-doses to cynomolgus monkeys, zintevir did not cause significant hemodynamic toxicity (unlike other oligonucleotides) at plasma drug concentrations that have shown anti-HIV activity *in vitro* (24,25).

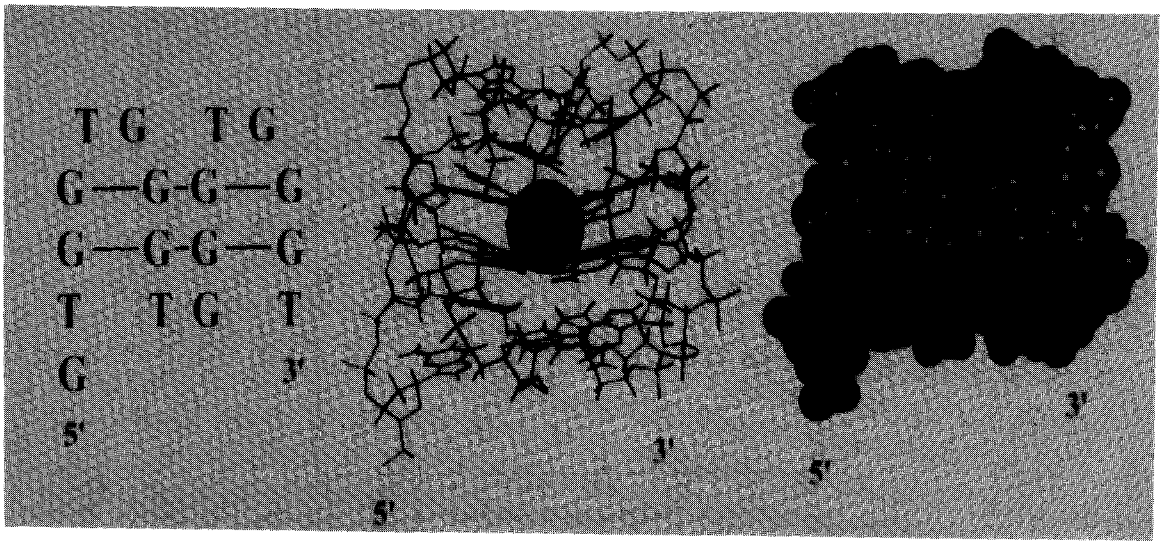

Fig. 18. Structure/model for the G-octet forming oligonucleotide T30177

Table 7. Anti-HIV activity of T30177

| Virus (strain) | Cell line | $EC_{50}$ (μM) | $CC_{50}$ (μM) | Selectivity index $CC_{50}/EC_{50}$ |
|---|---|---|---|---|
| HIV-1($III_B$) | CEM-SS | 2.83 | 92 | 32 |
| | MT-2 | 1.94 | 61 | 31 |
| | MT-4 | 0.15 | 70 | 466 |
| HIV-1(RF) | CEM-SS | 0.075 | 92 | 1226 |
| | MT-2 | 0.270 | 61 | 226 |
| | MT-4 | 0.037 | 70 | 1892 |
| HIV-2(ROD) | MT-4 | 27.5 | 70 | 2.5 |
| HIV-2(EHO) | MT-4 | 5.98 | 70 | 11.7 |
| SIV($MAC_{251}$) | MT-4 | 1.5 | 70 | 46 |

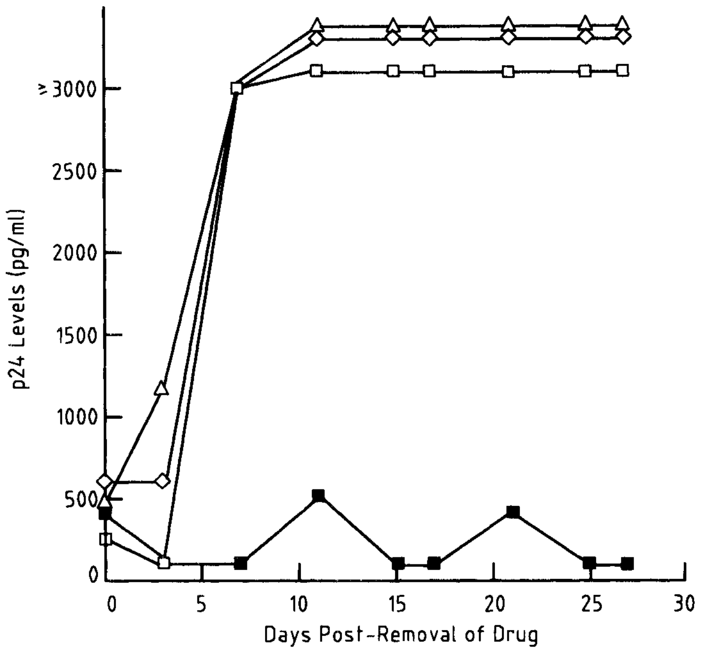

Fig. 19. Long-term suppression of HIV-1($III_B$) after treatment of infected cell cultures with T30177

MT-4 cells were infected with HIV-1(IIIB) at an MOI of 0.01 and were then cultured for 4 days in the presence of T30177 (■), AZT (□), DS 5000 (◊) or JM3100 (Δ) by using concentrations of drugs equivalent to 100-fold the respective $EC_{50}$ values. After 4 days the cells were washed extensively and were further incubated in drug-free medium. The level of viral p24 antigen in the culture medium was monitored at various times after removal of drug from the infected cell cultures.

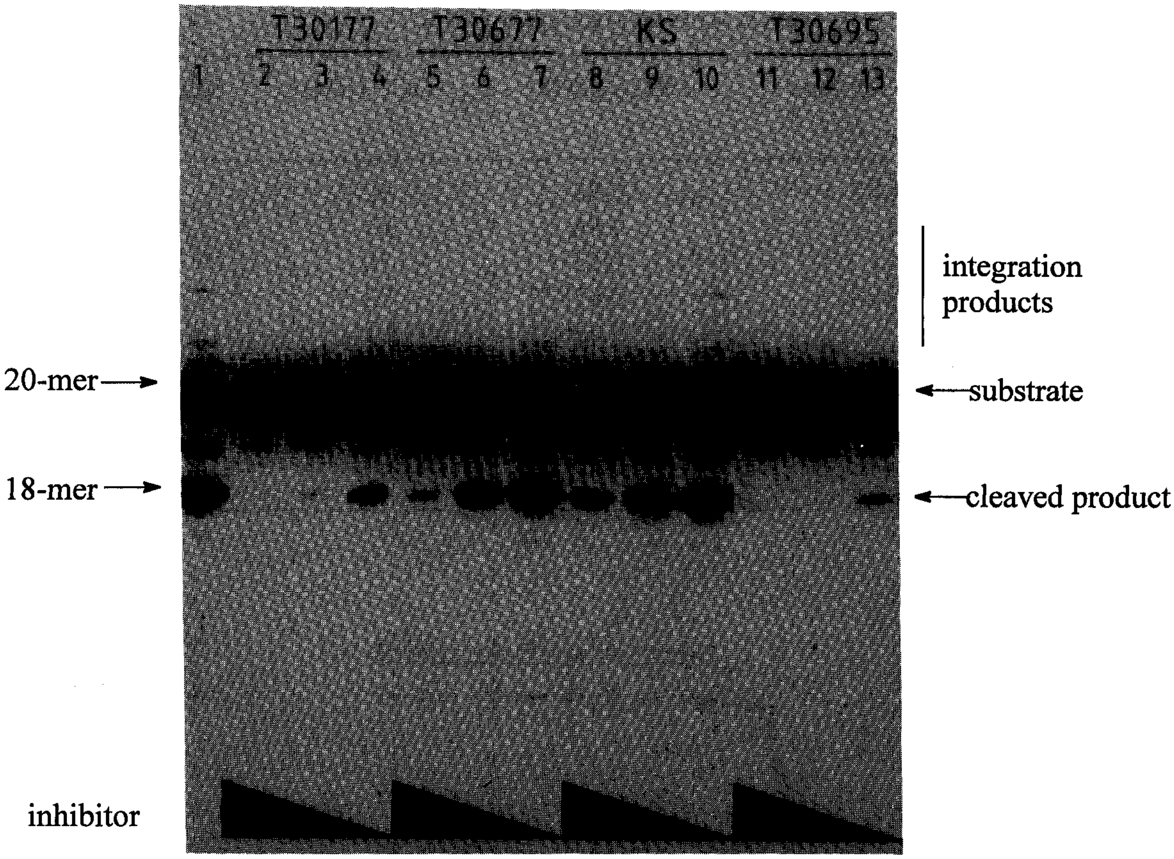

Fig. 20. Inhibition of the HIV-1 integrase by oligonucleotides

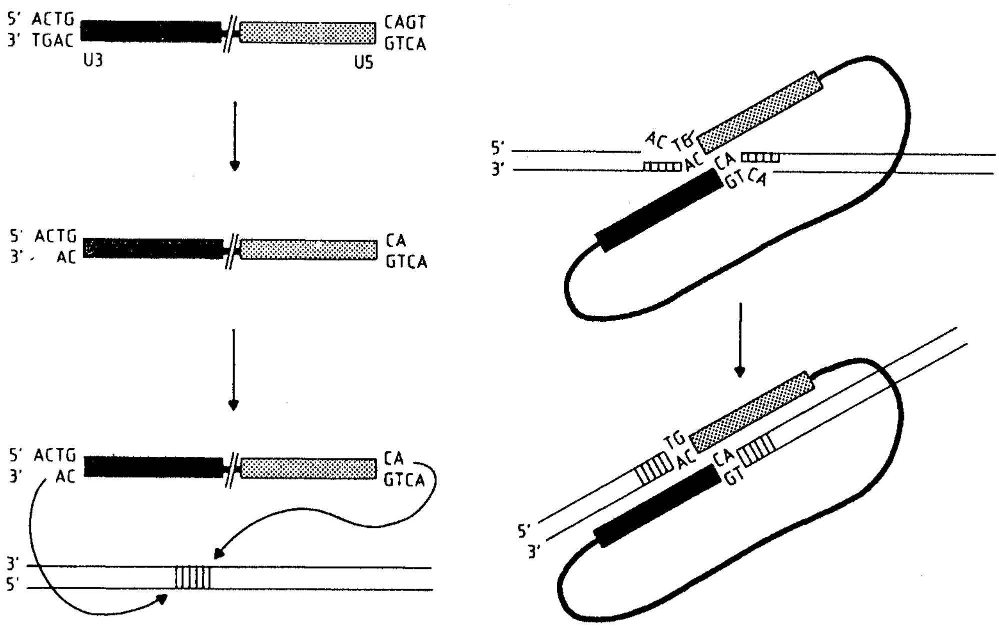

Fig. 21. Mechanism of HIV DNA integration

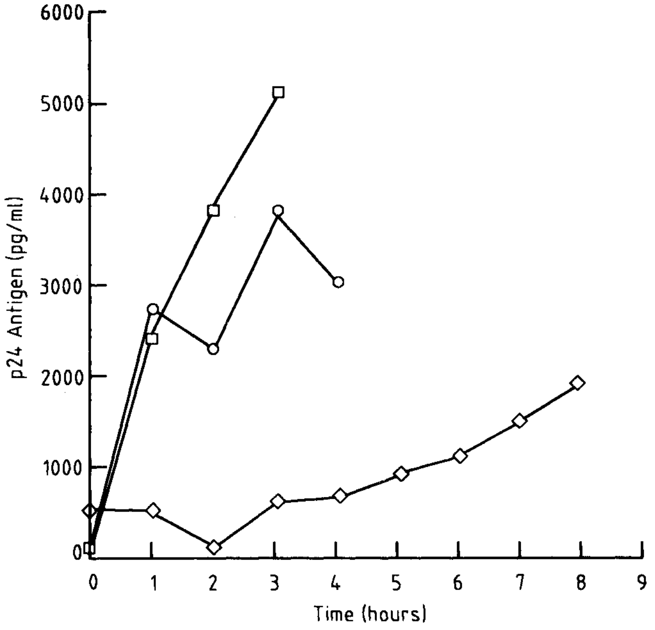

Fig. 22. Effect of time of drug addition on the inhibition profiles of T30177 (O), AZT (◊) and DS 5000 (□)

MT-4 cells infected with HIV-1(III$_B$) at an MOI of 1 were treated at various times during (time zero) or after virus infection with the test compounds at a concentration 100-fold greater than their respective $EC_{50}$ values. Viral p24 levels in the culture medium were monitored at 29 h postinfection.

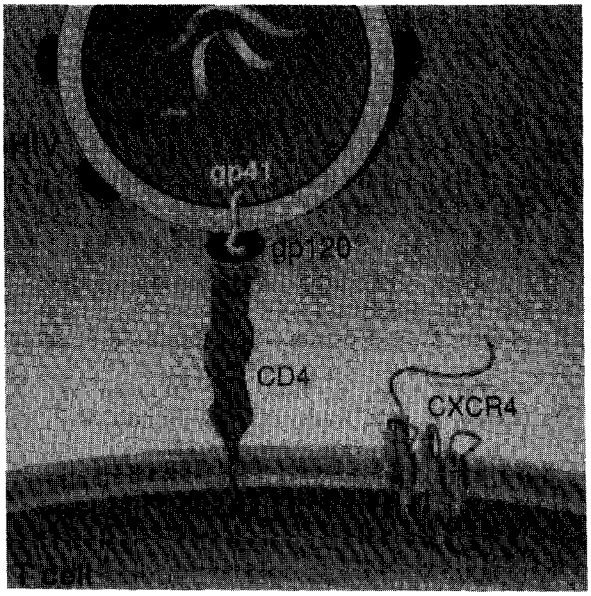

Fig. 23. Virus adsorption to the cells

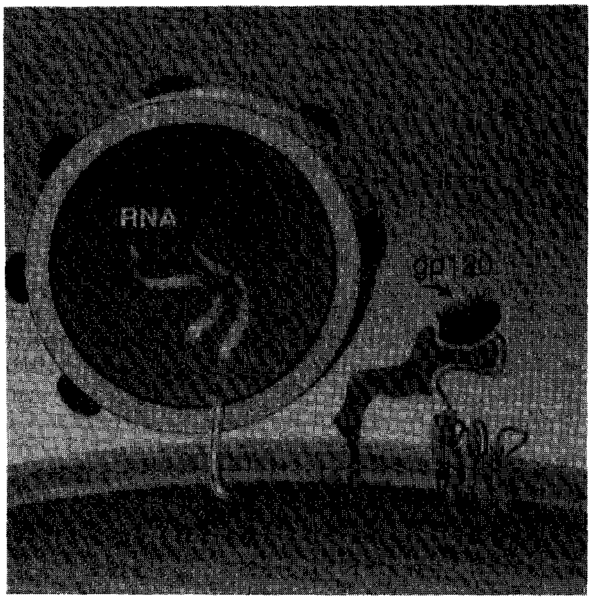

Fig. 24. Virus fusion with the cells

### Conclusion

To enter the cells, HIV must first interact through its viral envelope gp120 with the CD4 receptor of the host cell (virus adsorption) (Fig. 23) before the viral envelope can fuse with the outer cell membrane (Fig. 24). This virus cell fusion is made possible only after the viral envelope gp120 has also interacted with the second receptor [i.e. fusin (CXCR4)]. Our current investigations are aimed at elucidating the interactions of the compounds described here (polyanions, bicyclams, zintevir) with the viral envelope gp120 glycoprotein and the cellular receptors that are involved in the virus adsorption/fusion process.

### References

1. De Clercq, E. *Clin. Microbiol. Rev.* **8**: 200-239 (1995).
2. Witvrouw, M., Desmyter, J. & De Clercq, E. *Antiviral Chem. Chemother.* **5**: 345-359 (1994).
3. Yamamoto, N., Schols, D., De Clercq, E., Debyser, Z., Pauwels, R., Balzarini, J., Nakashima H., Baba, M., Hosoya, M., Snoeck, R., Neyts, J., Andrei, G., Murrer, B.A., Theobald, B., Bossard, G., Henson, G., Abrams, M. & Picker, D. *Mol. Pharmacol.* **42**: 1109-1117 (1992).
4. Song, R., Witvrouw, M., Schols, D., Robert, A., Balzarini, J., De Clercq, E., Bernadou, J. & Meunier, B. *Antiviral Chem. Chemother.*, in press (1997).
5. Esté, J.A., Schols, D., De Vreese, K., Van Laethem, K., Vandamme, A.-M., Desmyter, J. & De Clercq, E. *Mol. Pharmacol.*, submitted for publication (1997).
6. Ikeda, S., Neyts, J., Yamamoto, N., Murrer, B., Theobald, B., Bossard, G., Henson, G., Abrams, M., Picker, D. & De Clercq, E. *Antiviral Chem. Chemother.* **4**: 253-262 (1993).
7. Ikeda, S., Nishiya, S., Yamamoto, A., Yamase, T., Nishimura, C. & De Clercq, E. *Antiviral Chem. Chemother.* **5**: 47-50 (1994).
8. Ikeda, S., Neyts, J., Verma, S., Wickramasinghe, A., Mohan, P. & De Clercq, E. *Antimicrob. Agents Chemother.* **38**: 256-259 (1994).
9. Neyts, J. & De Clercq, E. *J. Acquir. Immun. Defic. Syndr. Hum. Retrovir.* **10**: 8-12.
10. De Clercq, E., Yamamoto, N., Pauwels, R., Baba, M., Schols, D., Nakashima, H., Balzarini, J., Debyser, Z., Murrer, B.A., Schwartz, D., Thornton, D., Bridger, G., Fricker, S., Henson, G., Abrams, M. & Picker, D. *Proc. Natl. Acad. Sci. USA* **89**: 5286-5290 (1992).
11. Bridger, G.J., Skerlj, R.T., Thornton, D., Padmanabhan, S., Martellucci, S.A., Henson, G.W., Abrams, M.J., Yamamoto, N., De Vreese, K., Pauwels, R. & De Clercq, E. *J. Med. Chem.* **38**, 366-378 (1995).
12. De Clercq, E., Yamamoto, N., Pauwels, R., Balzarini, J., Witvrouw, M., De Vreese, K., Debyser, Z., Rosenwirth, B., Peichl, P., Datema, R., Thornton, D., Skerlj, R., Gaul, F., Padmanabhan, S., Bridger, G., Henson, G. & Abrams, M. *Antimicrob. Agents Chemother.* **38**: 668-674 (1994).
13. De Clercq, E. *Int. J. Immunother.* **8**: 115-123 (1992).
14. De Clercq, E. *J. Med. Chem.* **38**: 2491-2517 (1995).
15. De Vreese, K., Reymen, D., Griffin, P., Steinkasserer, A., Werner, G., Bridger, G.J., Esté, J., James, W., Henson, G., Desmyter, J., Anné, J. & De Clercq, E. *Antiviral Res.* **29**: 209-219 (1996).
16. Esté, J.A., De Vreese, K., Witvrouw, M., Schmit, J.-C., Vandamme, A.-M., Anné, J., Desmyter, J., Henson, G.W., Bridger, G. & De Clercq, E. *Antiviral Res.* 29: 297-307 (1996).
17. De Vreese, K., Kofler-Mongold, V., Leutgeb, C., Weber, V., Vermeire, K., Schacht, S., Anné, J., De Clercq, E., Datema, R. & Werner, G. *J. Virol.* **70**: 689-696 (1996).
18. Datema, R., Rabin, L., Hincenbergs, M., Moreno, M.B., Warren, S., Linquist, V., Rosenwirth, B., Seifert, J. & McCune, J.M. *Antimicrob. Agents Chemother.* **40**: 750-754 (1996).
19. Bishop, J.S., Guf-Caffey, J.K., Ojwang, J.O., Smith, S.R., Hogan, M.E., Cossum, P.A., Rando, R.F. & Chaudhary, N. *J. Biol. Chem.* **271**: 5698-5703 (1996).
20. Ojwang, J.O., Buckheit, R.W., Pommier, Y., Mazumder, A., De Vreese, K., Esté, J.A., Reymen, D., Pallansch, L.A., Lackman-Smith, C., Wallace, T.L., De Clercq, E., McGrath, M.S. & Rando, R.F. *Antimicrob. Agents Chemother.* **39**: 2426-2435 (1995).
21. Mazumder, A., Neamati, N., Ojwang, J.O., Sunder, S., Rando, R.F. & Pommier, Y. *Biochemistry* **35**: 13762-13771 (1996).
22. Cherepanov, C., Esté, J.A., Rando, R.F., Ojwang, J.O., Reekmans, G., Steinfeld, R., David, G., De Clercq, E. & Debyser, Z. *Mol. Pharmacol.*, submitted for publication (1997).
23. Esté, J.A., Schols, D., De Vreese, K., Cherepanov, P., Witvrouw, M., Pannecouque, C., Debyser, Z., Rando, R.F., Desmyter, J. & De Clercq, E. *Mol. Pharmacol.*, submitted for publication (1997).
24. Wallace, T.L., Bazemore, S.A., Kornbrust, D.J. & Cossum, P.A. *J. Pharmacol. Exp. Ther.* **278**: 1306-1312 (1996).
25. Wallace, T.L., Bazemore, S.A., Kornbrust, D.J. & Cossum, P.A. *J. Pharmaco. Exp. Ther.* **278**: 1313-1317 (1996).

